# Suppression of IRAK1 or IRAK4 Catalytic Activity, but Not Type 1 IFN Signaling, Prevents Lupus Nephritis in Mice Expressing a Ubiquitin Binding–Defective Mutant of ABIN1

**DOI:** 10.4049/jimmunol.1600788

**Published:** 2016-11-02

**Authors:** Sambit K. Nanda, Marta Lopez-Pelaez, J. Simon C. Arthur, Francesco Marchesi, Philip Cohen

**Affiliations:** * Medical Research Council Protein Phosphorylation and Ubiquitylation Unit, University of Dundee, Dundee DD1 5EH, United Kingdom;; † Division of Immunology and Cell Signaling, School of Life Sciences, University of Dundee, Dundee DD1 5EH, United Kingdom; and; ‡ School of Veterinary Medicine, University of Glasgow, Glasgow G61 1QH, United Kingdom

## Abstract

Polymorphisms in the *TNIP1* gene encoding A20-binding inhibitor of NF-κB1 (ABIN1) predispose to lupus and other autoimmune diseases in at least eight human populations. We found previously that knock-in mice expressing a ubiquitin-binding–defective mutant of ABIN1 (ABIN1[D485N]) develop autoimmunity as they age and succumb to a disease resembling lupus nephritis in humans. In this article, we report that Flt3-derived dendritic cells from these mice overproduced type 1 IFNs upon stimulation with ligands that activate TLR7 or TLR9. However, crossing ABIN1[D485N] mice to IFNAR1-knockout mice that do not express the α-subunit of the type 1 IFNR did not prevent splenomegaly, the appearance of high serum levels of autoantibodies and other Igs, or liver inflammation and only reduced kidney inflammation modestly. In contrast, crossing ABIN1[D485N] mice to knock-in mice expressing catalytically inactive mutants of IRAK1 or IRAK4 prevented splenomegaly, autoimmunity, and liver and kidney inflammation. Our results support the notion that IRAK1 and/or IRAK4 are attractive targets for the development of drugs to prevent, and perhaps treat, lupus nephritis and other autoinflammatory diseases caused by the decreased ability of ABIN1 or other proteins to restrict the strength of MyD88 signaling.

## Introduction

The protein A20-binding inhibitor of NF-κB1 (ABIN1) is required to prevent systemic lupus erythematosus (SLE), because mutant mice expressing the functionally defective ABIN1[D485N] mutant, which cannot interact with ubiquitin chains, leads to the spontaneous development of autoimmunity (1). ABIN1[D485N] mice develop splenomegaly several months after birth. Their spleens contain far more germinal centers than those of wild-type (WT) mice, leading to elevated levels of Igs in the serum after 5–6 mo, which include Igs to host nuclear Ags and dsDNA. Immune complexes are deposited on the kidneys and on the blood vessels of the spleen and heart, leading to the recruitment of complement factors and severe inflammation and destruction of these organs (1). A similar phenotype was observed in ABIN1-knockout (KO) mice (2). Renal disease that develops spontaneously in ABIN1[D485N] mice is characterized by focal-to-diffuse proliferative glomerulonephritis resembling the class III and IV lupus nephritis observed in human SLE patients (3). Moreover, genome-wide association studies identified polymorphisms in *TNIP1*, the gene encoding ABIN1, which predispose to lupus, psoriasis, and other autoimmune disorders in eight human populations (4–13). Two polymorphisms were associated with reduced expression of ABIN1 protein (13). These findings, and the increased severity of the disease observed in female animals (1), suggest that the ABIN1[D485N]–knock-in mouse may be a good model for some forms of human lupus.

Enhanced expression of IFN-regulated genes, termed the IFN signature (14–17), was observed in a number of human patients with SLE, and Abs that neutralize IFN-α isotypes have entered clinical trials (18, 19). In this study, we found that the production of type 1 IFNs by plasmacytoid dendritic cells (pDCs) from ABIN1[D485N] mice was enhanced. This led us to cross them to IFNAR1-KO mice, a receptor essential for all known type 1 IFN signaling events. However, we found that crossing ABIN1[D485N] mice to IFNAR1-KO mice did not suppress the development of autoimmunity and only modestly reduced kidney inflammation. Therefore, we explored other possible ways in which autoimmunity might be prevented.

We showed previously that autoimmunity in ABIN1[D485N] mice is prevented by crossing them to MyD88-KO mice, establishing that hyperactivation of the MyD88 signaling network underlies the disease (1). In this pathway, the activation of TLRs recruits the adaptor molecule MyD88, which is followed by interaction of MyD88 with IRAK4 and then the interaction of IRAK4 with other IRAK family members to form an oligomeric complex termed the Myddosome (20, 21). Myddosome formation induces the formation of hybrid ubiquitin chains containing Lys^63^-linked and Met^1^-linked ubiquitin oligomers (22), which facilitate activation of the canonical IκB kinase (IKK) complex by the protein kinase TGF-β–activated kinase 1 (TAK1) (22, 23, reviewed in Ref. 24). The IKK complex activates the two critical transcription factors NF-κB and IRF5 (25, 26), whereas TAK1 also induces activation of the JNKs and p38 MAPKs, which switch on additional transcription factors and stimulate posttranscriptional events controlling the synthesis, processing, and secretion of inflammatory mediators. ABIN1 interacts with Lys^63^- and Met^1^-linked ubiquitin oligomers and is thought to compete with the TAK1 and IKK complexes for binding to the hybrid ubiquitin chains formed when this signaling pathway is activated. This explains why TAK1 and IKK are hyperactivated and the secretion of proinflammatory cytokines, such as IL-6, IL-12, and TNF-α, is enhanced in dendritic cells (DCs) expressing the ubiquitin-binding–defective ABIN1[D485N] mutant (1). These findings suggested that ABIN1[D485N] mice could be used to identify components of the MyD88 signaling pathway that might be attractive targets for the development of drugs to prevent and treat lupus. Therefore, we also crossed ABIN1[D485N] mice to mice expressing catalytically inactive mutants of IRAK1 (27) or IRAK4, as well as a functionally defective mutant of IRAK2 (28), the results of which are also reported in this article.

## Materials and Methods

### Generation and maintenance of mouse lines

ABIN1[D485N]–knock-in (1), IRAK2[E525A]–knock-in (28), and IRAK1[D359A]–knock-in (27) mice were described. ABIN1[D485N] mice were back-crossed to C57BL6/J mice (Jackson Laboratory) for up to 10 generations, IRAK1[D359A] mice up to 10 generations, and IRAK2[D525A] mice up to 8 generations. IRAK4[D329A]–knock-in mice were developed by Taconic-Artemis using embryonic stem cells from C57BL/6 mice and were further back-crossed to C57BL6/J mice for six generations. The C57BL/6 strain from Jackson Laboratory that was used for backcrossing these mice is free of the DOCK2 mutation that is reported to be present in C57BL mice from Harlan Laboratories and that affects the immune system (29). IFNAR1-KO mice (Crick Institute, London, U.K.) (30) were on a C57BL/6 background and were further backcrossed to C57BL6/J mice for six generations. Mice were maintained in individually ventilated cages under specific pathogen–free conditions, given free access to food and water, and housed in accordance with U.K. and European Union regulations. All procedures were carried out under a U.K. Home Office Project License and were subjected to local ethical review.

### TLR agonists

The TLR agonists Pam_3_CSK_4_ (TLR1/2), R848 (TLR7), CpG type A (ODN1585) (TLR9), and CpG type B (ODN1826) (TLR9) were from InvivoGen, and poly(dU) (TLR7) was from Sigma-Aldrich. Poly(dU) was added to the culture medium conjugated with Lipofectamine 2000 (Invitrogen), as described (28).

### Characterization of Flt3-derived DCs

The percentage of pDCs in Flt3-derived cell populations was assessed by staining with B220, PDCA1, CD11c, and CD11b Abs (Becton-Dickinson) and analyzed by flow cytometry.

### Abs

Abs recognizing p105/NFκB1 phosphorylated at Ser^933^, p38α MAPK phosphorylated at its Thr-Gly-Tyr motif, total p38α MAPK, total IRAK4, and GAPDH were from Cell Signaling Technology. An Ab recognizing IRAK4 phosphorylated at Thr^345^ and Ser^346^ was provided by V. Rao (Pfizer) (31). An Ab recognizing JNKs phosphorylated at their Thr-Pro-Tyr motif was from Invitrogen, and a rabbit secondary Ab conjugated to HRP was from Pierce.

### Histopathological analysis

Mice were euthanized by increasing CO_2_ levels. The kidneys and liver from each mouse were removed, fixed in 10% neutral buffered formalin for ≥48 h, and then embedded in paraffin. For liver, one sample from the left lobe and two from the median lobe were trimmed and processed. Sections (4 μm) were prepared, and liver and kidney sections were stained with H&E. Kidney sections were also stained with periodic acid–Schiff (PAS) reagent, and liver sections were stained with Sirius Red. Kidney and liver tissue sections were assessed by a veterinary pathologist (F.M.) who was blinded to the genotype of the mice in the different cohorts. The multiparametric semiquantitative scoring system used to assess renal and liver changes was based on the principles and guidelines for histopathologic scoring in research (32).

### Other methods

Generation and culture of bone marrow–derived macrophages (BMDMs) and Flt3-derived DCs, cell lysis, immunoblotting and cytokine measurements, cDNA synthesis, and RNA extraction were carried out as described (28). Quantitative PCR was performed as described (28), with the exception that SsoFast EvaGreen Supermix (Bio-Rad) was used. The primers used to measure *ifnb*, *ifna4*, and *ifna6* mRNA were described previously (33). Autoantibodies were measured by ELISA (1), and Ig isotypes were measured using a kit from Millipore.

### Statistical significance

Statistical significance was calculated using the two-tailed Student *t* test or Mann–Whitney *U* test with GraphPad Prism software. Additional details are given in the figure legends.

## Results

### Type 1 IFNs are overproduced in Flt3-derived DCs from ABIN1[D485N] mice

**FIGURE 1.**
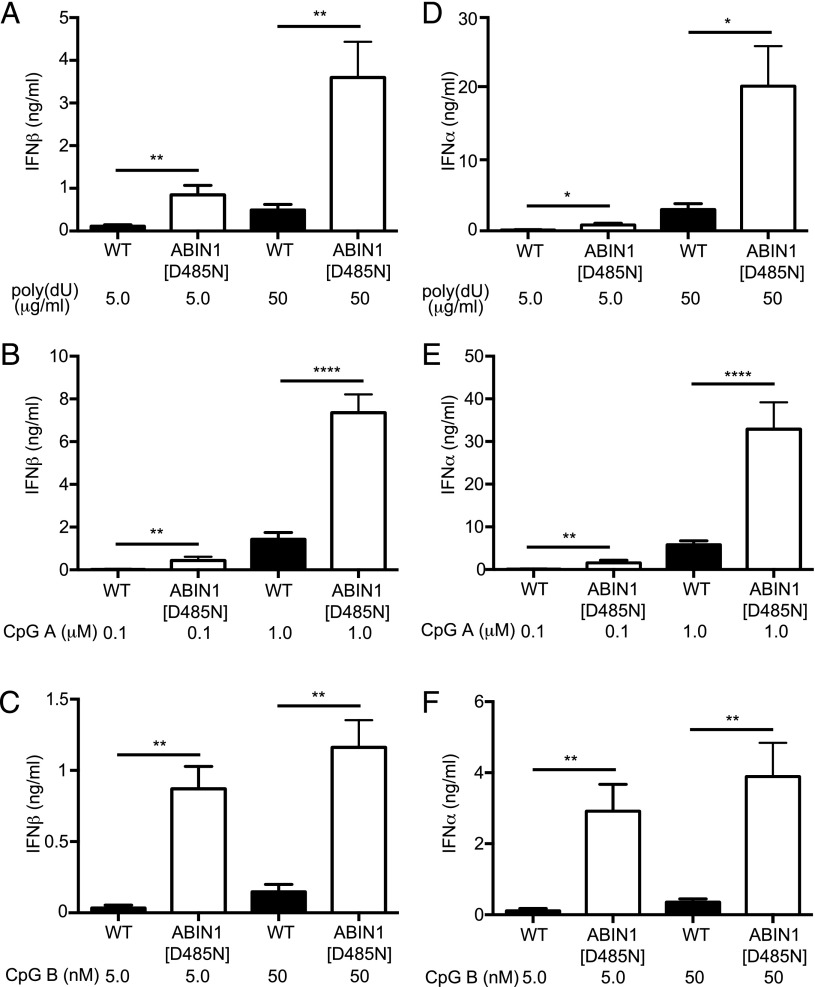
Enhanced IFN secretion in Flt3-derived DCs from ABIN1[D485N] mice. Flt3-derived DCs (3.5 × 10^5^ cells) from 6–8-wk-old WT mice or ABIN1[D485N] mice were stimulated for 12 h with the indicated concentrations of poly(dU) (**A** and **D**), CpG A (**B** and **E**), or CpG B (**C** and **F**). The concentrations of IFN-β (A–C) and IFN-α (D–F) in the cell culture medium was measured by ELISA using a mouse IFN-β kit (BioLegend) or VeriKine Mouse IFN Alpha ELISA Kit (PBL IFN Source), respectively. Error bars represent the mean ± SEM for four separate experiments carried out on cells from a total of 12 mice for each genotype. *p ≤ 0.05, **p ≤ 0.01, ****p ≤ 0.0001, Student *t* test.

**FIGURE 2.**
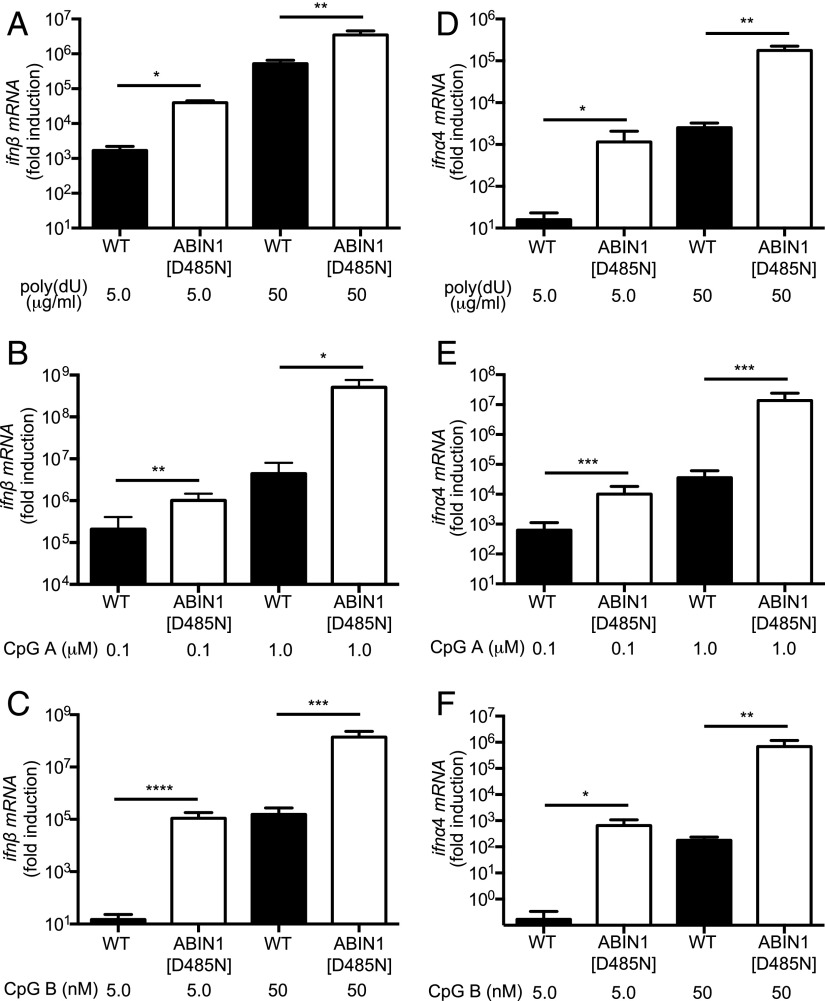
Enhanced *ifn-β* and *ifn-α* mRNA in Flt3-derived DCs from ABIN1[D485N] mice. Flt3-derived DCs (3.5 × 10^5^ cells) from 6–8-wk-old WT mice or ABIN1[D485N] mice were stimulated for 12 h with the indicated concentrations of poly(dU) (**A** and **D**), CpG A (**B** and **E**), or CpG B (**C** and **F**). The total RNA was extracted from the cells, and mRNA encoding *ifn-β* (A–C) or *ifna4* (D–F) was measured by quantitative RT-PCR. Error bars represent the mean ± SEM for four separate experiments carried out on cells from a total of 12 mice for each genotype. *p ≤ 0.05, **p ≤ 0.01, ***p ≤ 0.001, ****p ≤ 0.0001, Student *t* test.

pDCs are a major source of the type 1 IFNs produced by the MyD88 signaling network. Therefore, we compared IFN production in Flt3-derived DCs from ABIN1[D485N] and WT mice, which are widely used as a model for pDC function (34). We found that Flt3-derived DCs from ABIN1[D485N] mice secreted much higher levels of IFN-β (Fig. 1A–C) and IFN-α (Fig. 1D–F) and produced much higher levels of *ifnβ* (Fig. 2A–C), *ifn*α4 (Fig. 2D–F) and *ifnα6* mRNA (Supplemental Fig. 2) than pDCs from WT mice (note that the ordinates in Fig. 2 and Supplemental Fig. 2 are plotted on a log scale). Enhanced type 1 IFN production was not explained by an increased proportion of B220^+^ and PDCA^+^ pDCs in the preparations from ABIN1[D485N] mice. Indeed, the proportion of these cells relative to B220^−^ and PDCA^−^ cells was lower in preparations from these mice (27.76 ± 2.02% versus 37.18 ± 2.77% in WT mice). In contrast, the proportion of B220^−^ and PDCA^−^ cells increased from 4.8 ± 0.73% in WT mice to 6.1 ± 1.0% in ABIN1[D485N] mice. Taken together, our results demonstrate that ABIN1 restricts the production of type 1 IFNs in Flt3-derived DCs.

### Lupus nephritis in ABIN1[D485N] × IFNAR1-KO mice

**FIGURE 3.**
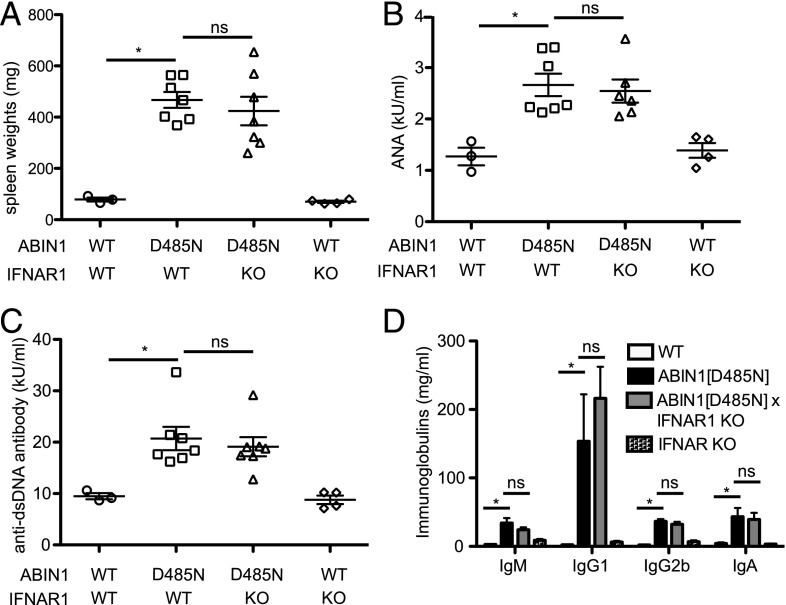
Hallmarks of autoimmunity are not reduced in ABIN1[D485N] × IFNAR1-KO mice. Three WT mice, six or seven ABIN1[D485N] mice, six or seven ABIN1[D485N] × IFNAR1-KO mice, and four IFNAR1-KO mice were used for all of the experiments. (**A**) Spleen weights of 6-mo-old WT mice (○), ABIN1[D485N]–knock-in mice (□), ABIN1[D485N] × IFNAR1-KO mice (△), and IFNAR1-KO mice (◇). Each symbol represents data from one mouse, and the horizontal lines show the average ± SEM. Total ANAs (**B**), anti-dsDNA Abs (**C**), and Ig isotypes (**D**) in the serum of 6-mo-old mice of the indicated genotypes were measured by ELISA. Each symbol represents one mouse, and the horizontal lines shows the average ± SEM. *p < 0.05, Mann–Whitney *U* test. ns, not significant.

**FIGURE 4.**
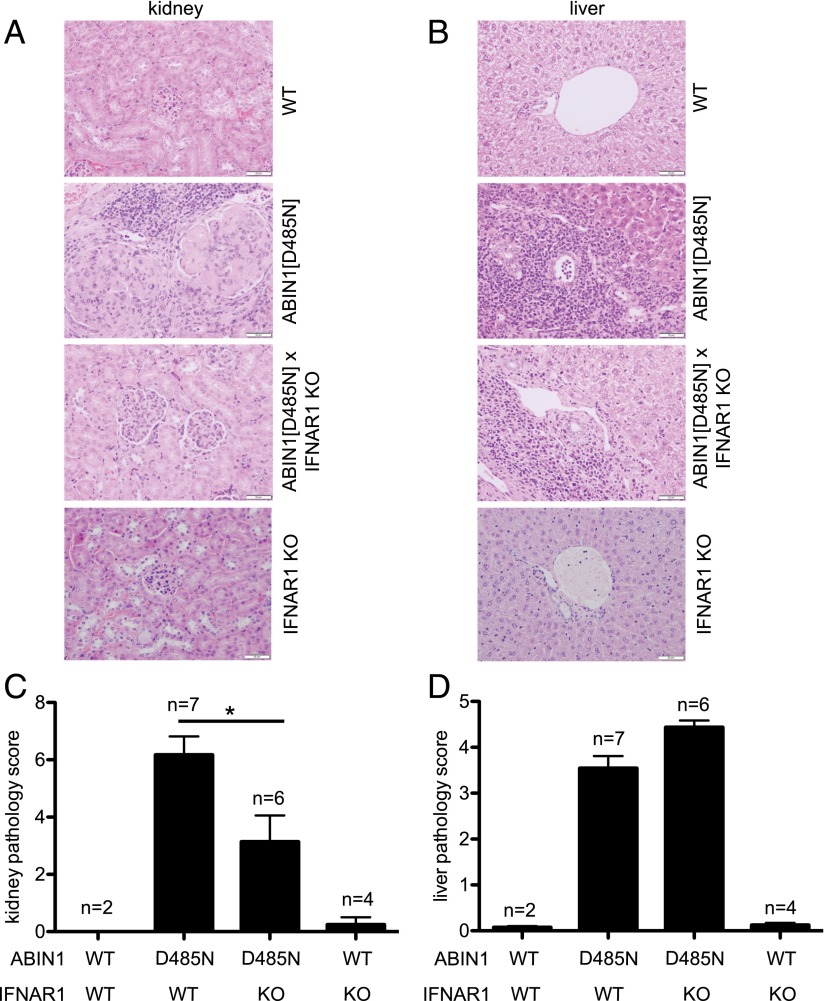
Reduced glomerulonephritis but unaffected liver inflammation in ABIN1[D485N] × IFNAR1-KO mice. Representative H&E staining of kidney (**A**) and liver (**B**) sections from 6-mo-old WT mice, ABIN1[D485N]–knock-in mice, ABIN1[D485N] × IFNAR1-KO mice, and IFNAR1-KO mice. Scale bars, 0.05 mm. Assessment of kidney (**C**) and liver (**D**) pathology scores (see *Materials and Methods*) from two WT mice, seven ABIN1[D485N] mice, six ABIN1[D485N × IFNAR1-KO mice, and four IFNAR1-KO mice. *p < 0.05, Mann–Whitney *U* test.

These experiments raised the question of whether the elevated levels of type 1 IFNs in ABIN[D485N] mice were responsible for the development of lupus nephritis in these animals. Therefore, we crossed them to *IFNAR1*-KO mice, a gene essential for every known signaling event triggered by type 1 IFNs. At 6 mo of age, ABIN1[D485N] × IFNAR1-KO mice showed enlarged spleens that were similar in size to those observed in ABIN1[D485N] mice (Fig. 3A), and the high serum levels of anti-nuclear Abs (ANAs), anti-self dsDNA, or other Ig isotypes in ABIN1[D485N] mice were not reduced in ABIN1[D485N] × IFNAR1-KO mice (Fig. 3B–D). In contrast, there was a partial reduction in the extent of glomerulonephritis in ABIN1[D485N] × IFNAR1-KO mice compared with ABIN1[D485N] mice (Fig. 4A, 4C). However, the liver inflammation found in ABIN1[D485N] mice at 6 mo of age was not reduced in ABIN1[D485N] × IFNAR1-KO mice (Fig. 4B, 4D).

### Lupus nephritis and liver inflammation in ABIN1[D485N] mice are prevented by crossing to mice expressing catalytically inactive mutants of IRAK1 or IRAK4

We showed previously that autoimmunity in ABIN1[D485N] mice is prevented by crossing to MyD88-KO mice (1). Because the other components of the Myddosome are IRAK1 and IRAK4 and the inactive pseudokinase IRAK2, we studied whether the loss of IRAK1 or IRAK4 catalytic activity, or IRAK2 function, prevented lupus in ABIN1[D485N] mice.

To investigate the importance of IRAK4 catalytic activity, we generated a knock-in mouse in which IRAK4 was replaced by the kinase-inactive IRAK4[D329A] mutant (Supplemental Fig. 1). This mutation prevents interaction of Asp^329^ with the magnesium ion of Mg-ATP without significantly affecting kinase conformation (35). Similar to mice expressing different kinase-inactive IRAK4 mutants (36, 37), IFN-β or IFN-α secretion induced by TLR ligation was undetectable in Flt3-derived DCs from IRAK4[D329A] mice (Supplemental Fig. 3A, 3B), and TNF-α, IL-6, and IL-12p40 secretion (Supplemental Fig. 3C–E) and MyD88 signaling (Fig. 3F, 3G) in BMDMs were also greatly reduced. IRAK4[D329A] mice, as well as the kinase-inactive IRAK1[D359A] mice (27), were then crossed to ABIN1[D485N] mice.

**FIGURE 5.**
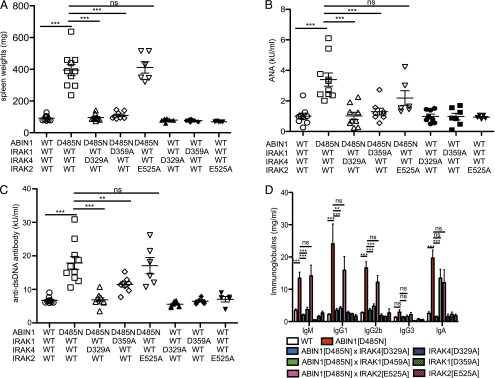
Autoimmunity in ABIN1[D485N] mice is prevented by crossing to mice expressing kinase-inactive mutants of IRAK4 or IRAK1. A total of 9 or 10 WT mice, 10 ABIN1[D485N] mice, 10 ABIN1[D485N] × IRAK4[D329A] mice, 8 ABIN1[D485N] × IRAK1[D359A] mice, 6 ABIN1[D485N] × IRAK2[E525A] mice, 7 or 8 IRAK4[D329A] mice, 7 or 8 IRAK1[D359A] mice, and 5 IRAK2[E525A] mice were used for all the experiments. (**A**) Spleen weights of 6-mo-old mice of the indicated genotypes. Each symbol represents one mouse, and the horizontal lines show the average ± SEM. Total ANAs (**B**), anti-dsDNA Abs (**C**), and Ig isotypes (**D**) in the serum of 24-wk-old mice of the indicated genotypes were measured by ELISA. (B and C) Each symbol represents one mouse, and the horizontal lines shows the average ± SEM. **p > 0.005, ***p < 0.005, Mann–Whitney *U* test. ns, nonsignificant.

**FIGURE 6.**
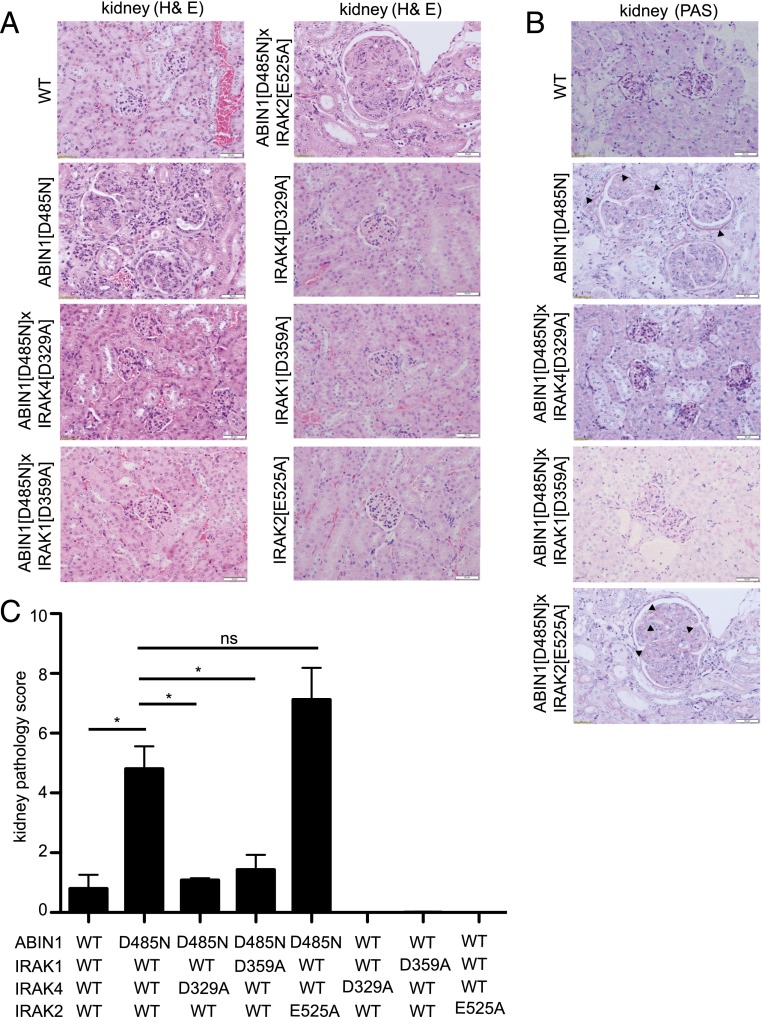
Glomerulonephritis in ABIN1[D485N] mice is suppressed by crossing to mice expressing kinase-inactive mutants of IRAK4 or IRAK1. Representative H&E staining (**A**) and PAS staining (**B**) of kidney sections from 6-mo-old WT mice, ABIN1[D485N]–knock-in mice, ABIN1[D485N] × IRAK4[D329A] mice, ABIN1[D485N] × IRAK1[D359A] mice, ABIN1[D485N] × IRAK2[E525A] mice, IRAK4[D329A] mice, IRAK1[D359A] mice, and IRAK2[E525A] mice. Scale bars, 0.05 mm. (B) The arrowheads show PAS^+^ material. (**C**) Assessment of kidney pathology scores (see *Materials and Methods*). All of the results shown were carried out using four mice of each genotype. *p > 0.05, Mann–Whitney *U* test. ns, not significant.

**FIGURE 7.**
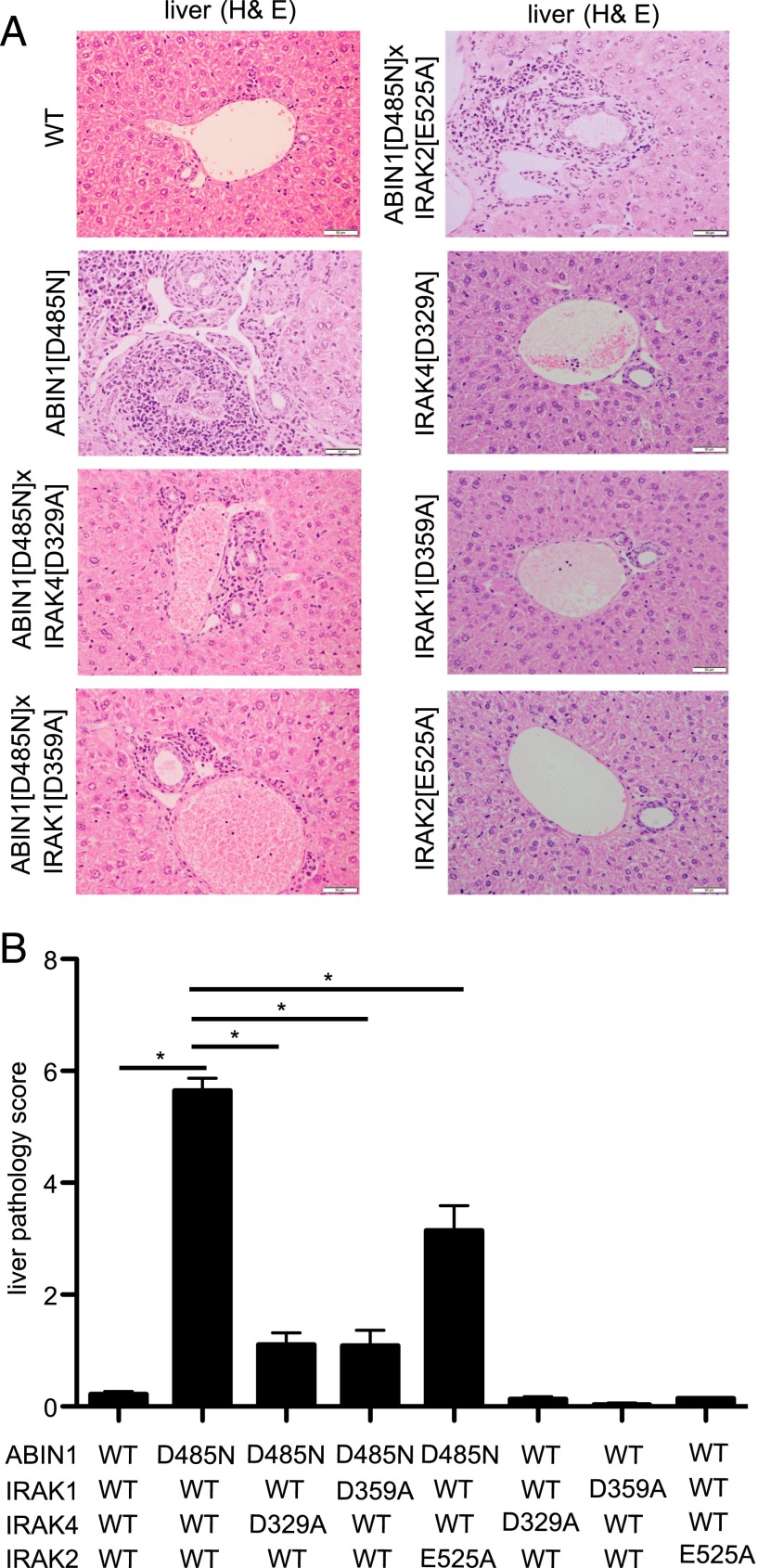
Liver inflammation in ABIN1[D485N] mice is reduced by crossing to mice expressing functionally inactive mutants of IRAK1, IRAK2, or IRAK4. (**A**) Representative H&E staining of liver sections from 6-mo-old WT mice, ABIN1[D485N]–knock-in mice, ABIN1[D485N] × IRAK4[D329A] mice, ABIN1[D485N] × IRAK1[D359A] mice, ABIN1[D485N] × IRAK2[E525A] mice, IRAK4[D329A] mice, IRAK1[D359A] mice, and IRAK2[E525A] mice. Scale bar, 0.05 mm. (**B**) Assessment of liver pathology scores (see *Materials and Methods*). All of the results shown were carried out using four mice of each genotype. *p > 0.05, Mann–Whitney *U* test.

Neither ABIN1[D485N] × IRAK4[D329A] mice nor ABIN1[D485N] × IRAK1[D359A] mice developed splenomegaly (Fig. 5A) and, consistent with these observations, the levels of ANAs (Fig. 5B), dsDNA Abs (Fig. 5C), and other IgGs and IgM (Fig. 5D) in the serum of these mice were similar to WT mice. However, the high level of IgA found in the serum of ABIN1[D485N] mice was suppressed by crossing to IRAK4[D329A] mice but not by crossing to IRAK1[D359A] mice (Fig. 5D). Moreover, no kidney inflammation was detectable in ABIN1[D485N] × IRAK4[D329A] mice or ABIN1[D485N] × IRAK1[D359A] mice at 6 mo of age (Fig. 6) (see *Materials and Methods* for scoring system), and the liver inflammation present in ABIN1[D485N]–knock-in mice was also largely reduced after crossing to IRAK4[D329A] or IRAK1[D359A] mice (Fig. 7B). Liver fibrosis along with inflammatory changes observed in ABIN1[D485N] mice were drastically reduced after crossing to IRAK4[D329A] or IRAK1[D359A] mice but were not reduced by crossing to IFNAR1-KO mice (Supplemental Fig. 4).

We also crossed ABIN1[D485N] mice to knock-in mice expressing the IRAK2[E525A] mutant, which is unable to interact with TRAF6. Proinflammatory cytokine production induced by several TLR agonists is greatly reduced in BMDMs from IRAK2[E525A]–knock-in mice (28). ABIN1[D485N] × IRAK2[E525A] mice still displayed splenomegaly and increased levels of autoantibodies and other Igs at 6 mo of age, similar to ABIN1[D485N] mice (Fig. 5). Moreover, the extent of kidney inflammation in ABIN1[D485N] × IRAK2[E525A] mice was similar to that in ABIN1[D485N] mice (Fig. 6). Interestingly, liver inflammation and attending fibrosis were partially reduced in ABIN1[D485N] × IRAK2[E525A] mice (Fig. 7, Supplemental Fig. 4).

## Discussion

Type 1 IFNs, which have important roles in regulating innate and adaptive immunity, also were implicated in the pathogenesis of lupus. However, we report that, although crossing ABIN1[D485N] mice to IFNAR1-KO mice modestly reduced kidney inflammation, it did not prevent the splenomegaly, elevated levels of Igs (including autoantibodies), and the liver pathology that occur spontaneously in ABIN1[D485N] mice (Figs. 3, 4, Supplemental Fig. 4). These findings indicate that elevated levels of IFN-regulated genes are not the primary cause of autoimmunity in ABIN1[D485N] mice and that enhanced IFN signaling induces the severity of glomerulonephritis by a mechanism that is independent of autoantibody production. Our findings suggest that SLE in patients expressing ABIN1 variants known to predispose to this disease may derive relatively little benefit from anti–type 1 IFN therapy. However, it cannot be excluded that the overproduction of type 2 and/or type 3 IFNs is also required to drive autoimmunity.

In contrast to our findings, studies reported that crossing two other lines of lupus-prone mice to IFNAR1-KO mice reduced the levels of autoantibodies and glomerulonephritis and, therefore, increased life span (38, 39). However, the mutations causing autoimmunity in these mice are unknown. Interestingly, in one of these lines, blockade of IL-6 also suppressed the development of autoimmunity (40, 41), indicating that elevated levels of more than one cytokine are required to trigger SLE. The lupus nephritis that develops in TANK-KO mice, and which is driven by hyperactivation of the MyD88 signaling network (42, 43), was similarly prevented by crossing to IL-6–KO mice (43). A recent study divided human SLE into a number of categories: some display elevated levels of IFN-regulated genes, whereas others have elevated levels of different inflammatory cytokines and chemokines (44). Taken together, these observations suggest that the effective treatment of SLE patients will require the development of a number of therapies, depending on the mutations that underlie the disease.

The most striking finding in the current study was that the autoimmune phenotype of ABIN1[D485N] mice was prevented by crossing to knock-in mice in which IRAK4 or IRAK1 was replaced by catalytically inactive mutants. These results are consistent with autoimmunity in ABIN1[D485N] mice being driven by hyperactivation of the MyD88 signaling network and raise the possibility that small molecule inhibitors of IRAK4 and/or IRAK1 prevent (and perhaps reverse) autoimmune diseases in which alterations in the *TNIP1* gene encoding ABIN1 (*Introduction*) or other genes causing hyperactivation of MyD88 signaling are a contributory factor. IRAK1 and IRAK4 do not participate in the cytoplasmic pathways by which viral RNA (RIGI, MDA5 pathways) or DNA (STING pathway) induce the production of type 1 IFNs (45, 46). Therefore, SLE patients treated with IRAK1 or IRAK4 inhibitors may have less risk for developing severe viral infection compared with patients given anti-IFN therapy. IRAK4-deficient children are susceptible to infection by pyogenic bacteria until they reach puberty, but they are not more susceptible to viral infection (47).

Although genome-wide association studies identified IRAK1 polymorphisms as a risk factor for SLE (48–50), to our knowledge the current study is the first to provide evidence that specific inhibition of IRAK1 kinase may have therapeutic potential for the prevention of SLE. Such inhibitors might have significant advantages compared with IRAK4-specific inhibitors or dual inhibitors of IRAK1 and IRAK4, which are likely to impair the MyD88-dependent production of proinflammatory cytokines more drastically than the loss of IRAK1 catalytic activity alone. For example, the production of proinflammatory cytokines by several TLR agonists was found to be unimpaired in BMDMs from IRAK1[D359A] mice (28). In contrast, the production of type 1 IFNs and proinflammatory cytokines by ligands that activate the MyD88 signaling network specifically was virtually abolished in BMDMs from IRAK4[D329A]–knock-in mice (Supplemental Fig. 3), as well as in knock-in mice expressing different catalytically inactive mutants of IRAK4 (36, 37). Therefore, patients treated with an IRAK1-specific inhibitor might have a reduced risk for infection by microbial pathogens than patients treated with an IRAK4 inhibitor.

## Supplementary Material

Data Supplement

## References

[1] Nanda S. K. Venigalla R. K. Ordureau A. Patterson-Kane J. C. Powell D. W. Toth R. Arthur J. S. Cohen P. 2011 Polyubiquitin binding to ABIN1 is required to prevent autoimmunity. J. Exp. Med. 208: 1215–1228. 21606507 10.1084/jem.20102177 PMC3173241

[2] Zhou J. Wu R. High A. A. Slaughter C. A. Finkelstein D. Rehg J. E. Redecke V. Häcker H. 2011 A20-binding inhibitor of NF-κB (ABIN1) controls Toll-like receptor-mediated CCAAT/enhancer-binding protein β activation and protects from inflammatory disease. Proc. Natl. Acad. Sci. USA 108: E998–E1006. 22011580 10.1073/pnas.1106232108 PMC3207663

[3] Caster D. J. Korte E. A. Nanda S. K. McLeish K. R. Oliver R. K. G’Sell R. T. Sheehan R. M. Freeman D. W. Coventry S. C. Kelly J. A. 2013 ABIN1 dysfunction as a genetic basis for lupus nephritis. J. Am. Soc. Nephrol. 24: 1743–1754. 23970121 10.1681/ASN.2013020148 PMC3810087

[4] Nair R. P. Duffin K. C. Helms C. Ding J. Stuart P. E. Goldgar D. Gudjonsson J. E. Li Y. Tejasvi T. Feng B. J. Collaborative Association Study of Psoriasis 2009 Genome-wide scan reveals association of psoriasis with IL-23 and NF-kappaB pathways. Nat. Genet. 41: 199–204. 19169254 10.1038/ng.311 PMC2745122

[5] Gateva V. Sandling J. K. Hom G. Taylor K. E. Chung S. A. Sun X. Ortmann W. Kosoy R. Ferreira R. C. Nordmark G. 2009 A large-scale replication study identifies TNIP1, PRDM1, JAZF1, UHRF1BP1 and IL10 as risk loci for systemic lupus erythematosus. Nat. Genet. 41: 1228–1233. 19838195 10.1038/ng.468 PMC2925843

[6] Han J. W. Zheng H. F. Cui Y. Sun L. D. Ye D. Q. Hu Z. Xu J. H. Cai Z. M. Huang W. Zhao G. P. 2009 Genome-wide association study in a Chinese Han population identifies nine new susceptibility loci for systemic lupus erythematosus. Nat. Genet. 41: 1234–1237. 19838193 10.1038/ng.472

[7] Bossini-Castillo L. Martin J. E. Broen J. Simeon C. P. Beretta L. Gorlova O. Y. Vonk M. C. Ortego-Centeno N. Espinosa G. Carreira P. Spanish Scleroderma Group 2013 Confirmation of TNIP1 but not RHOB and PSORS1C1 as systemic sclerosis risk factors in a large independent replication study. Ann. Rheum. Dis. 72: 602–607. 22896740 10.1136/annrheumdis-2012-201888 PMC3887516

[8] Gregersen P. K. Kosoy R. Lee A. T. Lamb J. Sussman J. McKee D. Simpfendorfer K. R. Pirskanen-Matell R. Piehl F. Pan-Hammarstrom Q. 2012 Risk for myasthenia gravis maps to a (151) Pro→Ala change in TNIP1 and to human leukocyte antigen-B*08. Ann. Neurol. 72: 927–935. 23055271 10.1002/ana.23691 PMC3535539

[9] Kawasaki A. Ito S. Furukawa H. Hayashi T. Goto D. Matsumoto I. Kusaoi M. Ohashi J. Graham R. R. Matsuta K. 2010 Association of TNFAIP3 interacting protein 1, TNIP1 with systemic lupus erythematosus in a Japanese population: a case-control association study. Arthritis Res. Ther. 12: R174. 20849588 10.1186/ar3134 PMC2991001

[10] Munir S. ber Rahman S. Rehman S. Saba N. Ahmad W. Nilsson S. Mazhar K. Naluai Å. T. 2015 Association analysis of GWAS and candidate gene loci in a Pakistani population with psoriasis. Mol. Immunol. 64: 190–194. 25481369 10.1016/j.molimm.2014.11.015

[11] Shi Y. Jia Y. Hou S. Fang J. Zhou Y. Kijlstra A. Yang P. 2014 Association of a TNIP1 polymorphism with Vogt-Koyanagi-Harada syndrome but not with ocular Behcet’s disease in Han Chinese. PLoS One 9: e95573. 24788730 10.1371/journal.pone.0095573 PMC4008420

[12] Lessard C. J. Li H. Adrianto I. Ice J. A. Rasmussen A. Grundahl K. M. Kelly J. A. Dozmorov M. G. Miceli-Richard C. Bowman S. UK Primary Sjögren’s Syndrome Registry 2013 Variants at multiple loci implicated in both innate and adaptive immune responses are associated with Sjögren’s syndrome. Nat. Genet. 45: 1284–1292. 24097067 10.1038/ng.2792 PMC3867192

[13] Adrianto I. Wang S. Wiley G. B. Lessard C. J. Kelly J. A. Adler A. J. Glenn S. B. Williams A. H. Ziegler J. T. Comeau M. E. BIOLUPUS and GENLES Networks 2012 Association of two independent functional risk haplotypes in TNIP1 with systemic lupus erythematosus. Arthritis Rheum. 64: 3695–3705. 22833143 10.1002/art.34642 PMC3485412

[14] Becker A. M. Dao K. H. Han B. K. Kornu R. Lakhanpal S. Mobley A. B. Li Q. Z. Lian Y. Wu T. Reimold A. M. 2013 SLE peripheral blood B cell, T cell and myeloid cell transcriptomes display unique profiles and each subset contributes to the interferon signature. PLoS One 8: e67003. 23826184 10.1371/journal.pone.0067003 PMC3691135

[15] Bennett L. Palucka A. K. Arce E. Cantrell V. Borvak J. Banchereau J. Pascual V. 2003 Interferon and granulopoiesis signatures in systemic lupus erythematosus blood. J. Exp. Med. 197: 711–723. 12642603 10.1084/jem.20021553 PMC2193846

[16] Baechler E. C. Batliwalla F. M. Karypis G. Gaffney P. M. Ortmann W. A. Espe K. J. Shark K. B. Grande W. J. Hughes K. M. Kapur V. 2003 Interferon-inducible gene expression signature in peripheral blood cells of patients with severe lupus. Proc. Natl. Acad. Sci. USA 100: 2610–2615. 12604793 10.1073/pnas.0337679100 PMC151388

[17] Crow M. K. Kirou K. A. Wohlgemuth J. 2003 Microarray analysis of interferon-regulated genes in SLE. Autoimmunity 36: 481–490. 14984025 10.1080/08916930310001625952

[18] Merrill J. T. Wallace D. J. Petri M. Kirou K. A. Yao Y. White W. I. Robbie G. Levin R. Berney S. M. Chindalore V. Lupus Interferon Skin Activity (LISA) Study Investigators 2011 Safety profile and clinical activity of sifalimumab, a fully human anti-interferon α monoclonal antibody, in systemic lupus erythematosus: a phase I, multicentre, double-blind randomised study. Ann. Rheum. Dis. 70: 1905–1913. 21798883 10.1136/ard.2010.144485

[19] Chuntharapai A. Lai J. Huang X. Gibbs V. Kim K. J. Presta L. G. Stewart T. A. 2001 Characterization and humanization of a monoclonal antibody that neutralizes human leukocyte interferon: a candidate therapeutic for IDDM and SLE. Cytokine 15: 250–260. 11594789 10.1006/cyto.2001.0934

[20] Motshwene P. G. Moncrieffe M. C. Grossmann J. G. Kao C. Ayaluru M. Sandercock A. M. Robinson C. V. Latz E. Gay N. J. 2009 An oligomeric signaling platform formed by the Toll-like receptor signal transducers MyD88 and IRAK-4. J. Biol. Chem. 284: 25404–25411. 19592493 10.1074/jbc.M109.022392 PMC2757241

[21] Lin S. C. Lo Y. C. Wu H. 2010 Helical assembly in the MyD88-IRAK4-IRAK2 complex in TLR/IL-1R signalling. Nature 465: 885–890. 20485341 10.1038/nature09121 PMC2888693

[22] Emmerich C. H. Ordureau A. Strickson S. Arthur J. S. Pedrioli P. G. Komander D. Cohen P. 2013 Activation of the canonical IKK complex by K63/M1-linked hybrid ubiquitin chains. Proc. Natl. Acad. Sci. USA 110: 15247–15252. 23986494 10.1073/pnas.1314715110 PMC3780889

[23] Zhang J. Clark K. Lawrence T. Peggie M. W. Cohen P. 2014 An unexpected twist to the activation of IKKβ: TAK1 primes IKKβ for activation by autophosphorylation. Biochem. J. 461: 531–537. 24911653 10.1042/BJ20140444 PMC4206954

[24] Clark K. Nanda S. Cohen P. 2013 Molecular control of the NEMO family of ubiquitin-binding proteins. Nat. Rev. Mol. Cell Biol. 14: 673–685. 23989959 10.1038/nrm3644

[25] Lopez-Pelaez M. Lamont D. J. Peggie M. Shpiro N. Gray N. S. Cohen P. 2014 Protein kinase IKKβ-catalyzed phosphorylation of IRF5 at Ser462 induces its dimerization and nuclear translocation in myeloid cells. Proc. Natl. Acad. Sci. USA 111: 17432–17437. 25326418 10.1073/pnas.1418399111 PMC4267347

[26] Ren J. Chen X. Chen Z. J. 2014 IKKβ is an IRF5 kinase that instigates inflammation. Proc. Natl. Acad. Sci. USA 111: 17438–17443. 25326420 10.1073/pnas.1418516111 PMC4267374

[27] Goh E. T. Arthur J. S. Cheung P. C. Akira S. Toth R. Cohen P. 2012 Identification of the protein kinases that activate the E3 ubiquitin ligase Pellino 1 in the innate immune system. Biochem. J. 441: 339–346. 22007846 10.1042/BJ20111415

[28] Pauls E. Nanda S. K. Smith H. Toth R. Arthur J. S. Cohen P. 2013 Two phases of inflammatory mediator production defined by the study of IRAK2 and IRAK1 knock-in mice. J. Immunol. 191: 2717–2730. 23918981 10.4049/jimmunol.1203268 PMC3849919

[29] Mahajan V. S. Demissie E. Mattoo H. Viswanadham V. Varki A. Morris R. Pillai S. 2016 Striking immune phenotypes in gene-targeted mice are driven by a copy-number variant originating from a commercially available C57BL/6 strain. Cell Rep. 15: 1901–1909. 27210752 10.1016/j.celrep.2016.04.080 PMC4892502

[30] Redford P. S. Mayer-Barber K. D. McNab F. W. Stavropoulos E. Wack A. Sher A. O’Garra A. 2014 Influenza A virus impairs control of *Mycobacterium tuberculosis* coinfection through a type I interferon receptor-dependent pathway. J. Infect. Dis. 209: 270–274. 23935205 10.1093/infdis/jit424 PMC3873785

[31] Cushing L. Stochaj W. Siegel M. Czerwinski R. Dower K. Wright Q. Hirschfield M. Casanova J. L. Picard C. Puel A. 2014 Interleukin 1/Toll-like receptor-induced autophosphorylation activates interleukin 1 receptor-associated kinase 4 and controls cytokine induction in a cell type-specific manner. J. Biol. Chem. 289: 10865–10875. 24567333 10.1074/jbc.M113.544809 PMC4036451

[32] Gibson-Corley K. N. Olivier A. K. Meyerholz D. K. 2013 Principles for valid histopathologic scoring in research. Vet. Pathol. 50: 1007–1015. 23558974 10.1177/0300985813485099 PMC3795863

[33] Pauls E. Shpiro N. Peggie M. Young E. R. Sorcek R. J. Tan L. Choi H. G. Cohen P. 2012 Essential role for IKKβ in production of type 1 interferons by plasmacytoid dendritic cells. J. Biol. Chem. 287: 19216–19228. 22511786 10.1074/jbc.M112.345405 PMC3365954

[34] Naik S. H. Sathe P. Park H. Y. Metcalf D. Proietto A. I. Dakic A. Carotta S. O’Keeffe M. Bahlo M. Papenfuss A. 2007 Development of plasmacytoid and conventional dendritic cell subtypes from single precursor cells derived in vitro and in vivo. Nat. Immunol. 8: 1217–1226. 17922015 10.1038/ni1522

[35] Knighton D. R. Zheng J. H. Ten Eyck L. F. Ashford V. A. Xuong N. H. Taylor S. S. Sowadski J. M. 1991 Crystal structure of the catalytic subunit of cyclic adenosine monophosphate-dependent protein kinase. Science 253: 407–414. 1862342 10.1126/science.1862342

[36] Kim T. W. Staschke K. Bulek K. Yao J. Peters K. Oh K. H. Vandenburg Y. Xiao H. Qian W. Hamilton T. 2007 A critical role for IRAK4 kinase activity in Toll-like receptor-mediated innate immunity. J. Exp. Med. 204: 1025–1036. 17470642 10.1084/jem.20061825 PMC2118590

[37] Kawagoe T. Sato S. Jung A. Yamamoto M. Matsui K. Kato H. Uematsu S. Takeuchi O. Akira S. 2007 Essential role of IRAK-4 protein and its kinase activity in Toll-like receptor-mediated immune responses but not in TCR signaling. J. Exp. Med. 204: 1013–1024. 17485511 10.1084/jem.20061523 PMC2118579

[38] Agrawal H. Jacob N. Carreras E. Bajana S. Putterman C. Turner S. Neas B. Mathian A. Koss M. N. Stohl W. 2009 Deficiency of type I IFN receptor in lupus-prone New Zealand mixed 2328 mice decreases dendritic cell numbers and activation and protects from disease. J. Immunol. 183: 6021–6029. 19812195 10.4049/jimmunol.0803872 PMC2766036

[39] Santiago-Raber M. L. Baccala R. Haraldsson K. M. Choubey D. Stewart T. A. Kono D. H. Theofilopoulos A. N. 2003 Type-I interferon receptor deficiency reduces lupus-like disease in NZB mice. J. Exp. Med. 197: 777–788. 12642605 10.1084/jem.20021996 PMC2193854

[40] Finck B. K. Chan B. Wofsy D. 1994 Interleukin 6 promotes murine lupus in NZB/NZW F1 mice. J. Clin. Invest. 94: 585–591. 8040314 10.1172/JCI117373 PMC296134

[41] Mihara M. Takagi N. Takeda Y. Ohsugi Y. 1998 IL-6 receptor blockage inhibits the onset of autoimmune kidney disease in NZB/W F1 mice. Clin. Exp. Immunol. 112: 397–402. 9649207 10.1046/j.1365-2249.1998.00612.x PMC1904997

[42] Clark K. Takeuchi O. Akira S. Cohen P. 2011 The TRAF-associated protein TANK facilitates cross-talk within the IkappaB kinase family during Toll-like receptor signaling. Proc. Natl. Acad. Sci. USA 108: 17093–17098. 21949249 10.1073/pnas.1114194108 PMC3193242

[43] Kawagoe T. Takeuchi O. Takabatake Y. Kato H. Isaka Y. Tsujimura T. Akira S. 2009 TANK is a negative regulator of Toll-like receptor signaling and is critical for the prevention of autoimmune nephritis. Nat. Immunol. 10: 965–972. 19668221 10.1038/ni.1771 PMC2910115

[44] Banchereau R. Hong S. Cantarel B. Baldwin N. Baisch J. Edens M. Cepika A. M. Acs P. Turner J. Anguiano E. 2016 Personalized immunomonitoring uncovers molecular networks that stratify lupus patients. [Published erratum appears in 2016 *Cell* 165: 1548–1550] Cell 165: 551–565. 27040498 10.1016/j.cell.2016.03.008 PMC5426482

[45] Goubau D. Deddouche S. Reis e Sousa C. 2013 Cytosolic sensing of viruses. Immunity 38: 855–869. 23706667 10.1016/j.immuni.2013.05.007 PMC7111113

[46] Paludan S. R. Bowie A. G. 2013 Immune sensing of DNA. Immunity 38: 870–880. 23706668 10.1016/j.immuni.2013.05.004 PMC3683625

[47] Picard C. Casanova J. L. Puel A. 2011 Infectious diseases in patients with IRAK-4, MyD88, NEMO, or IκBα deficiency. Clin. Microbiol. Rev. 24: 490–497. 21734245 10.1128/CMR.00001-11 PMC3131061

[48] Jacob C. O. Zhu J. Armstrong D. L. Yan M. Han J. Zhou X. J. Thomas J. A. Reiff A. Myones B. L. Ojwang J. O. 2009 Identification of IRAK1 as a risk gene with critical role in the pathogenesis of systemic lupus erythematosus. Proc. Natl. Acad. Sci. USA 106: 6256–6261. 19329491 10.1073/pnas.0901181106 PMC2669395

[49] Sánchez E. Comeau M. E. Freedman B. I. Kelly J. A. Kaufman K. M. Langefeld C. D. Brown E. E. Alarcón G. S. Kimberly R. P. Edberg J. C. 2011 Identification of novel genetic susceptibility loci in African American lupus patients in a candidate gene association study. Arthritis Rheum. 63: 3493–3501. 21792837 10.1002/art.30563 PMC3205224

[50] Kaufman K. M. Zhao J. Kelly J. A. Hughes T. Adler A. Sanchez E. Ojwang J. O. Langefeld C. D. Ziegler J. T. Williams A. H. 2013 Fine mapping of Xq28: both *MECP2* and *IRAK1* contribute to risk for systemic lupus erythematosus in multiple ancestral groups. Ann. Rheum. Dis. 72: 437–444. 22904263 10.1136/annrheumdis-2012-201851 PMC3567234

