# Supplementary material for: Suppression of IRAK1 or IRAK4 Catalytic Activity, but Not Type 1 IFN Signaling, Prevents Lupus Nephritis in Mice Expressing a Ubiquitin Binding–Defective Mutant of ABIN1
Source: J Immunol. 2016 Nov 2;197(11):4266–73. doi: 10.4049/jimmunol.1600788 (PMC5114882; doi:10.4049/jimmunol.1600788)
Supplement: Data Supplement [file JI_1600788.zip › JI_1600788_Supplemental_Figures_1.pdf]

# Figure S1

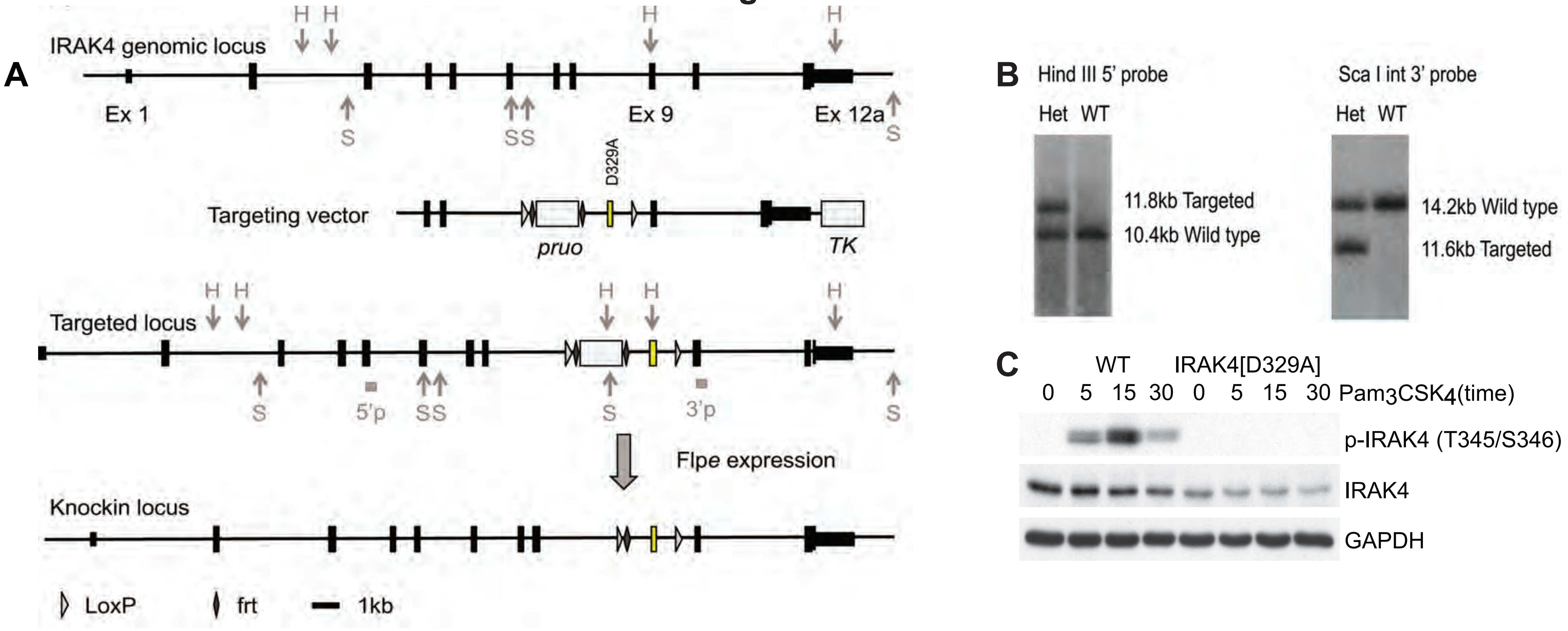


**Figure S1. Generation of IRAK4[D329A] knock-in mice.** IRAK4[D329A] knock-in mice were generated by Taconic Artemis by mutating Asp329 to Ala in the "DFG" motif of IRAK4, which is encoded in exon 9 of the mouse gene. This mutation does not affect overall kinase conformation significantly. The targeting strategy is shown in (**A**). The targeting vector contained the required mutation in exon 9 and additional loxP sites were introduced in the introns adjacent to this exon. Puromycin resistance (puro) and thymidine kinase (TK) cassettes were included for positive and negative selection, respectively. The sequence of the targeting vector is available on request. The vector was electroporated into C57Bl/6 ES cells and, following both positive and negative selection; resistant clones were screened by PCR Correct targeting was confirmed by Southern blot analysis using 5' and 3' probes on Hind III and Sca I-digested DNA, respectively. The location of the probes and the Hind III (H) and Sca I (S) sites are shown in (A) and representative Southern blots shown in (**B**). Correctly targeted ES cells were injected into blastocysts to generate germline-transmitting chimeric mice, which were crossed to C57Bl/6 Flpe transgenic mice to remove the puro gene and the IRAK4 allele bred away from the Flpe transgene in subsequent generations. Mouse genotyping was carried out by PCR of ear biopsy samples using GGCTGACATGTTCCAAGTCC and TACCCCACAGTTCACTGAAGC as primers for the IRAK4 gene. These primers amplify the region where the 5' loxP site is inserted and generate a wild type band of 163bp and a knock-in band of 305bp. The presence of the Flpe transgene was determined with the primers GGCAGAAGCACGCTTATCG and GACAAGCGTTAGTAGGCACAT, giving a 343bp band for samples with the transgene. (**C**) BMDM from IRAK4[D329A] and wild type mice were stimulated for the times indicated with 1.0 μg/ml Pam3CSK4 which activates the TLR1/2 heterodimer. The cells were lysed and cell extract (20 μg protein) subjected to SDS-PAGE and immunoblotting with antibodies that recognize all forms of IRAK4 or IRAK4 phosphorylated at Thr345 and Ser346. GAPDH was used as a loading control.

# Figure S2

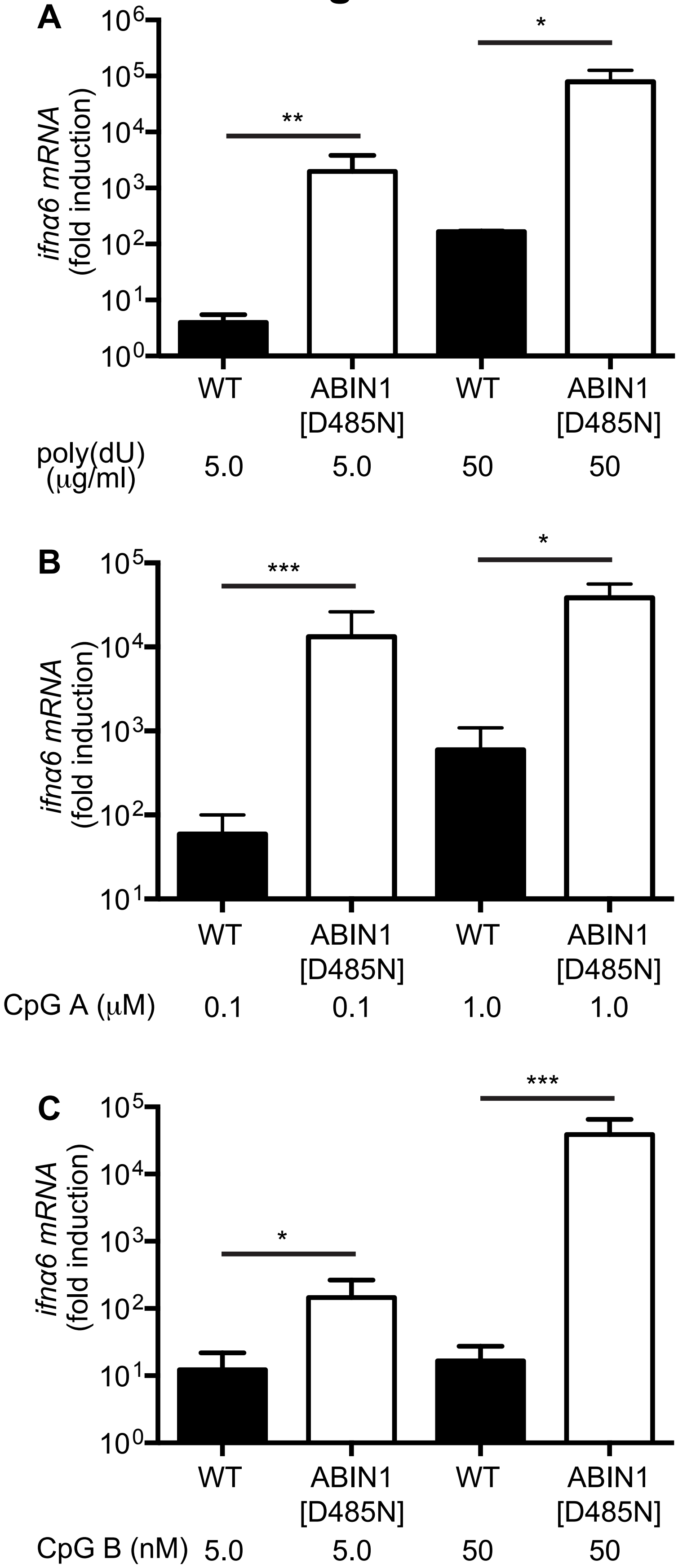


**Figure S2. Enhanced *ifna6* mRNA in Flt3-derived DCs from ABIN1[D485N] mice.**
(**A-C**) Flt3-derived DCs (3.5x105 cells) from 6-8 week old wild type (WT) mice (filled bars) or ABIN1[D485N] mice (open bars) were stimulated for 12 h with the indicated concentrations of poly(dU) (**A**), CpG A (**B**) or CpG B (**C**). The total RNA was extracted from the cells and mRNA encoding *ifna6* was quantitated by qRT-PCR. Error bars represent the mean ± SEM for four separate experiments carried out on cells from a total of 12 mice for each genotype.. * P< 0.05, ** P< 0.01, *** P< 0.001, ****P<0.0001 (Student's t-test).

# Figure S3

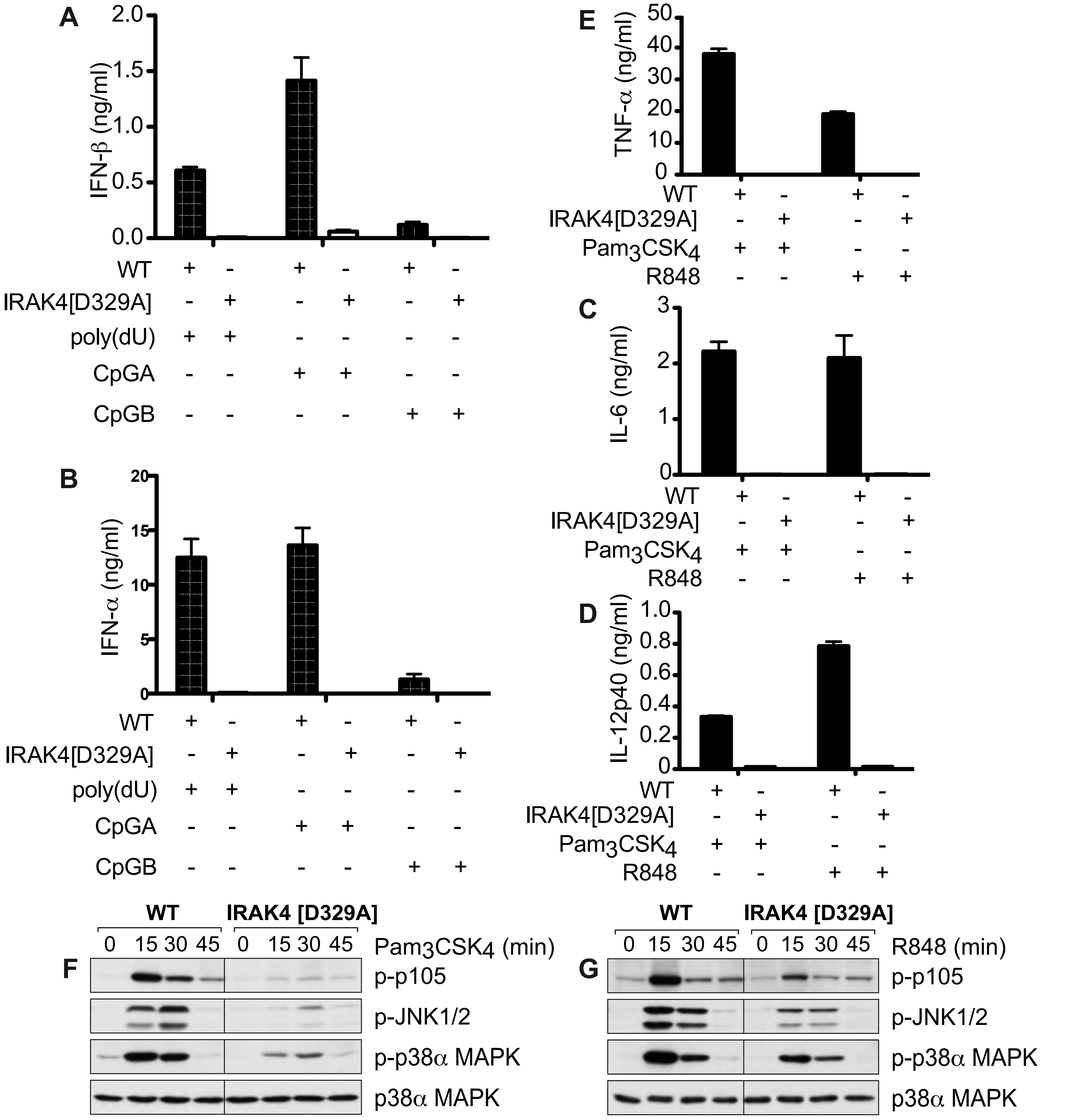


**Figure S3. TLR-induced signalling and cytokine production in IRAK4[D329A] mice.**
(**A, B**) Flt3-derived DCs (3.5x105 cells) from 6-8 week old WT or IRAK4[D329A] mice were incubated for 1 h in 96-well plates, then stimulated for 12 h with 50 μg/ml of the TLR7 agonist poly(dU), or with the TLR9 agonists CpG A (1.0 μM) CpG B (or 0.05 μM) and the concentrations of IFNβ andIFNα in the cell culture medium were measured by ELISA using the mouse IFNβ kit (Biolegend) or Verikine Mouse IFNα kit (PBL Interferon Source), respectively. (**C-E**). BMDM (5 x 105 cells) from 6-8 week old WT mice or IRAK4[D329A] mice were re-plated into 12-well tissue culture plates then stimulated for 8 h with 1.0 μg/ml Pam3CSK4 or 1.0 μg/ml of the TLR7 agonist R848. The concentrations of IL-6, IL-12p40 and TNF-α secreted into the cell culture medium were measured by ELISA (28). The error bars in A-E represent the mean ± SEM for experiments from three mice of each genotype. Similar results were obtained in two independent experiments. (**F, G**) BMDM from 6-8 week old IRAK4[D329A] or wild-type (WT) mice were stimulated for the times indicated with either 1.0 μg/ml Pam3CSK4 (1μg/ml) (**F**) or 1.0 μg/ml of R848 (**G**). Cell extract (20 μg protein) was subjected to SDS/PAGE and immunoblotted with the phospho-specific antibodies indicated. An antibody that recognises all forms of p38α MAP kinase was used as a loading control.

# Figure S4

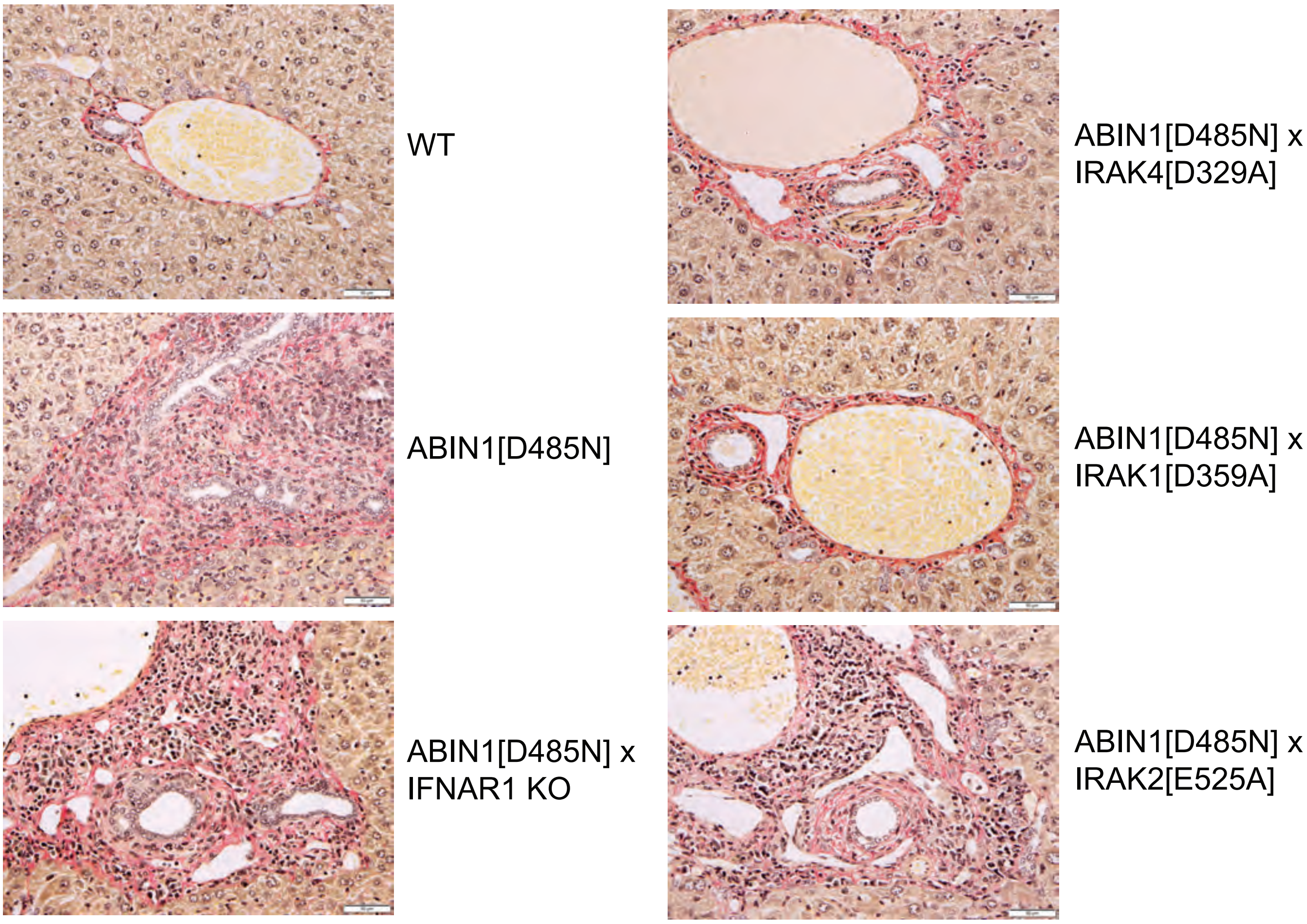


**Figure S4: Liver fibrosis in ABIN1[D485N] mice is reduced by crossing to mice expressing functionally inactive mutants of IRAK1, IRAK2 and IRAK4.**
Representative Sirius Red stained liver sections from six-month old wild type mice (WT), ABIN1[D485N] knock-in mice, ABIN1[D485N] X IFNAR1 KO mice, ABIN1[D485N] x IRAK4[D329A] mice, ABIN1[D485N] x IRAK1[D359A] mice, ABIN1[D485N] x IRAK2[E525A] mice (scale bar, 0.05 mm). Similar observations were made with four mice of each genotype.
